# The Epidemiology of Celiac Disease in the General Population and High-Risk Groups in Arab Countries: A Systematic Review

**DOI:** 10.1155/2020/6865917

**Published:** 2020-06-03

**Authors:** Ashraf El-Metwally, Paivi Toivola, Khalid AlAhmary, Salwa Bahkali, Ali AlKhathaami, Munira K. AlSaqabi, Shatha A. Al Ammar, Munazza Jawed, Saleh M. Alosaimi

**Affiliations:** ^1^College of Public Health and Health Informatics, King Saud Bin Abdulaziz University for Health Sciences, Riyadh, Saudi Arabia; ^2^King Abdullah Specialist Children's Hospital, King Abdulaziz Medical City, Riyadh, Saudi Arabia; ^3^Princess Nourah Bint Abdulrahman University, King Abdullah Bin AbdulAziz University Hospital, Riyadh, Saudi Arabia; ^4^King Abdulaziz Medical City, National Guard Health Affairs, College of Medicine, King Saud Bin Abdulaziz University for Health Sciences, Riyadh, Saudi Arabia; ^5^College of Medicine, King Saud Bin AbdulAziz University for Health Sciences, Riyadh, Saudi Arabia; ^6^King Abdullah Bin Abdulaziz University Hospital, Riyadh, Saudi Arabia; ^7^Dow University of Health Sciences, Karachi, Pakistan; ^8^King Abdulaziz Medical City, National Guard Health Affairs, King Saud Bin Abdulaziz University for Health Sciences, Riyadh, Saudi Arabia

## Abstract

**Background and Aims:**

Celiac disease (CD) is possibly the most common autoimmune disorder, which may lead to dietary problems in the Arab region. This paper is aimed at exploring the epidemiology of the celiac disease in Arab countries, including its prevalence, associated risk factors, and clinical patterns.

**Methods:**

An extensive search of the literature was conducted from electronic databases such as PubMed, Embase, and Google Scholar. In total, 134 research papers were retrieved. We extracted studies published from January 1996 to December 2019. Our search was limited to studies published in English. *Findings*. The review included 35 studies with 22,340 participants from 12 countries and demonstrated a wide variation in the prevalence of CD. The highest prevalence among the general population (3.2%) was reported in Saudi Arabia, and the lowest (0.1%) was reported in Tunisia. Women demonstrated a higher prevalence of celiac disease relative to men. The peak age at diagnosis fell between 1 and 3 years and 9-10 years. Most studies focused on type 1 diabetes. Children with type 1 diabetes have a higher prevalence of CD (range from 5.5% to 20%), while the prevalence of CD in Down's syndrome patients was 1.1% and 10.7% in UAE and Saudi Arabia, respectively. Other autoimmune diseases associated with CD are thyroid disease and irritable bowel disease. The most widely recognized clinical presentation was an inability to flourish and poor weight gain, followed by short stature, abdominal pain, abdominal distension, bloating, and chronic diarrhea.

**Conclusion:**

The prevalence of the celiac disease in Arab countries varies with sex and age. However, we found that celiac disease presented similar clinical characteristics independent of the geographic region. Longitudinal population-based studies are needed to better identify the true burden and determinants of celiac disease.

## 1. Introduction

Celiac disease (CD) is a chronic inflammatory disease of the upper small intestine triggered by gluten protein intolerance, which is prevalent in “genetically predisposed individuals.” Gluten is the wheat grain protein richly consumed in Western countries with an average daily intake of 10 to 20 grams/person/day [1]. It is comprised of prolamin and glutelin proteins. Both proteins abundantly possess glutamine and proline residues, which defy gastrointestinal digestion and promote the deamination process through the tissue transglutaminase (tTG) enzyme [1, 2]. It may lead to mucosal inflammation and villous atrophy, thus causing malabsorption [2]. The classical manifestation of CD is often present with all related signs and symptoms of malabsorption. Moreover, patients also experience diarrhea, steatorrhea, and loss of weight or growth failure [3]. Meanwhile, in children, the classical characteristics are diarrhea, failure to thrive, muscle wasting, poor appetite, abdominal distension, and sometimes emotional distress and lethargy [3].

The European Society for Paediatric Gastroenterology, Hepatology, and Nutrition (ESPGHAN) suggested diagnostic criteria for CD. According to the guidelines, it depends upon the gluten-dependent symptoms, CD-specific antibody levels, HLA-DQ2 and/or HLA-DQ8, and histopathologic findings, which are villous atrophy and crypt hyperplasia, in a biopsy of the duodenum [4].

Some epidemiological studies demonstrate that the prevalence of celiac disease has been undervalued, affecting not just Europeans but also the population of Mediterranean countries, including those in the Middle East [5, 6], where its prevalence of celiac disease is quite similar to that of the Western states [7]. The incidence of celiac diseases in the Middle East is reported to be high both among at-risk groups and the general population. This is due to dietary habits such as excessive consumption of barley and wheat as well as due to a higher frequency of DR3-DQ2 haplotypes [8]. From a global perspective, the incidence of celiac disease differs from 1 : 132 in Switzerland to 1 : 1000 and 1 : 2000 in other European countries [9]. In the risk groups for celiac disease, a hereditary connection is present in first-degree relatives with a frequency of 1 : 22 and in relatives of a second degree with a frequency of 1 : 39 [10]. Celiac disease is also prevalent in the Nordic countries [11] with a frequency in the population, estimated by the results of serological screening of blood donors (in some cases supplemented with biopsy), of about 1/300 of that in the rest of Europe (especially that of Ireland and Italy) and around 1/250 of some areas of the United States [12]. In some regions, the prevalence of the celiac disease is 1/100. The disease develops in relatives of the first degree of relationship with a frequency of 10-20% [13]. The ratio of occurrence classified based on gender is 2 : 1 (females to males). Several studies have identified risk groups in which celiac disease is detected most often [13, 14]. This group includes people suffering from other autoimmune and genetic diseases, including autoimmune thyroiditis, autoimmune liver disease, diabetes mellitus, Down's syndrome, and Turner syndrome, and relatives of celiac disease patients. The frequency of celiac disease in the risk groups can reach up to 10%. Therefore, an in-depth examination of patients with these diseases is recommended.

Several studies have been conducted about celiac disease in Arab countries; however, no recent epidemiological systematic review exists. We believed that ethnicity and geographical differences might affect disease frequency. Therefore, we carried out a systematic review to find the epidemiology of CD in the Arab countries, and this includes the prevalence, associated risk factors, and clinical features.

## 2. Methods

This study used a systematic review research approach. To locate primary studies relevant to our review, a systematic and comprehensive search of multiple electronic databases, such as PubMed, Embase, and Google Scholar, was performed, with each database searched individually. The keywords based on Medical Subject Headings (MeSH) such as celiac disease, Arab countries, Saudi Arabia, epidemiology, prevalence, and Middle Eastern countries were systematically applied line by line and replicated in every source database using Boolean operators: (celiac or coeliac) AND (Algeria or Bahrain or Egypt or Iraq or Jordan or Kuwait or Lebanon or Libya or Morocco or Mauritania or Oman or Palestine or Qatar or Saudi Arabia or Somalia or Sudan or Syria or Tunisia or United Arab Emirates or Yemen) AND (epidemiology or risk or burden or prevalence or incidence or impact or prognosis). Complete local journal searching and cross-referencing were undertaken by two reviewers who agreed on the final selection of the articles.

### 2.1. Inclusion and Exclusion Criteria

The inclusion criteria were original research studies published in peer-reviewed journals, mainly focusing on epidemiology, burden, prevalence, risk factors, incidence, or prognosis of celiac disease in Arab countries. Studies that utilized observational, retrospective, and prospective studies and were published in English between 1996 and 2019 were included. Studies that were published before 1996 focused on non-Arab populations, or enclosed case reports, case series, and quasiexperimental research designs were excluded from this review.

### 2.2. Study Selection

The titles/abstracts of the search outcomes were studied, and when the suitability of the articles was in question, the full-text articles were demanded and evaluated. Based on the exclusion and inclusion criteria, relevant full-text articles were assessed, screened, and reviewed by two researchers for inclusion. Any disagreements between authors were resolved through discussion with the third author. This ensured that only the articles relevant to the research questions were included. In total, 134 research papers were retrieved. Of these, 35 were deemed suitable for analysis and relevant for inclusion into our documented review by both reviewers.

### 2.3. Data Analysis

**Figure 1:**
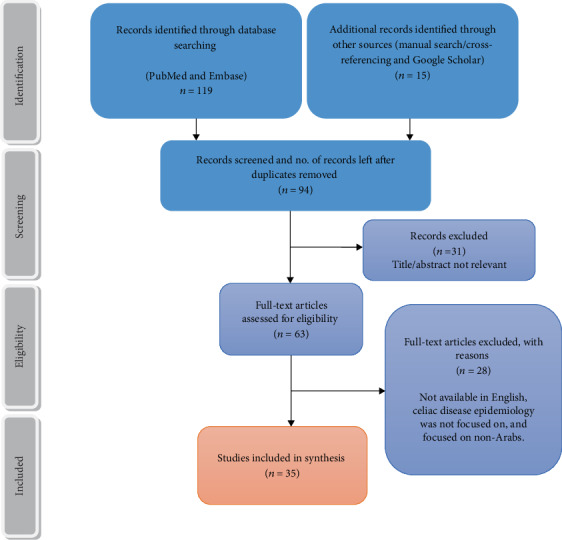
Retrieval of articles and screening process.

All analyses and reviews on literature were conducted based on the PRISMA “(Preferred Reporting Items for Systematic Reviews and Meta-Analyses) guidelines. Relevant extracted papers were synthesized systematically. The collected data was summarized through narrative with an overview of geographical location, study design, study settings, populations, sample sizes, and case definition. It was then followed by synthesis of the selected studies based on the outcome measures. Due to the heterogeneity of the presented data, a meta-analysis was not possible.  Figure 1 represents the article screening and retrieval process.

### 2.4. Quality Assessment

The quality of the studies included in this review was assessed using the Newcastle-Ottawa Scale (NOS) [15]. The scoring scale of the modified NOS ranges between 0 and 8: low-quality studies with NOS scores 0-2, medium quality studies with NOS scores 3-5, and high-quality studies with 6-8/9 NOS scores.

## 3. Results

Overall, 134 articles were reviewed and assessed for eligibility to meet our inclusion criteria. After individually reviewing each abstract against a prespecified inclusion criterion, 99 articles were excluded. This yielded 35 research articles, which focused primarily on the epidemiology of the celiac disease in Arab countries. The study selection process is summarized in Figure 1. We extracted articles from the period of 1996 to 2019. The studies included different research designs such as cross-sectional studies, prospective studies, case-control categorized under observational study design, and retrospective hospital-based studies with 22,340 participants from 12 Arab countries.

As seen in the following table, there were 35 published studies about celiac disease conducted in the Arab world. The national focus of these studies is divided in the following way: 17 were from Saudi Arabia, 1 from Algeria, 1 from Libya, 3 from Tunisia, 2 from Egypt, 2 from Oman, 2 from Jordan, 2 from the United Arab Emirates, 1 from Iraq, 1 from Kuwait, 1 from Qatar, 1 from Morocco, and 1 focused on the entire Middle East. The 22 studies focused on the prevalence, risk factors, and frequency of celiac disease among high-risk groups, and 9 focused on the prevalence of CD among the general population, while 4 of the 35 studies reported the clinical patterns and manifestation of CD.

### 3.1. Prevalence of Celiac Diseases among High-Risk Groups

It is clear from Table 1 that different researches have indicated that celiac disease presents an increased prevalence among several geographic regions. Most of the studies focused on type 1 diabetes. Children with type 1 diabetes have a higher prevalence of CD (range from 5.5% to 20%), while the prevalence of CD in Down's syndrome patients was 1.1% and 10.7% in UAE and Saudi Arabia, respectively. Other autoimmune diseases associated with CD are thyroid disease and irritable bowel disease.

### 3.2. Prevalence of CD among General Population

The prevalence of CD in healthy adult populations was found to range from 0.14% to 3.2%, the highest (3.2%) prevalence being reported in Saudi Arabia and the lowest (0.14%) in Tunisia (see Table 2). In healthy children, the estimated prevalence ranged from 0.6% to 1.5%. Studies conducted in Saudi Arabia estimated the frequency of the disease to be 1 : 250-100. Approximately, the peak of diagnosis falls around the age of 1 to 3 years.

### 3.3. Clinical Pattern of Celiac Disease

Table 3 shows the clinical characteristics of laboratory-confirmed CD patients. The most widely recognized presentation was an inability to flourish and poor weight gain, followed by short stature, abdominal pain, abdominal distension, bloating, and chronic diarrhea.

## 4. Discussion

This review included 35 studies, showing a wide variation in the prevalence of CD, ranging from 0.14% to 3.2%. The highest prevalence among otherwise healthy individuals was reported in Saudi Arabia at 3.2%, and the lowest was in Tunisia at 0.14%. Gender distribution revealed a high occurrence in females. The peak age at diagnosis fell around the age of 1-3 years to 9-10 years. It was also found to be associated with type 1 diabetes in Saudi children in addition to thyroid disease, Down syndrome, and irritable bowel disease. The most common symptoms were an inability to flourish, poor weight gain, short stature, chronic diarrhea, abdominal pain, gas, and bloating. Most of the studies used anti-tTG titers and EMA for their diagnosis. The role of family history was also highlighted in one study. Moreover, a gluten-free diet was found to improve laboratory parameters. However, noncompliance for this was also picked up by one of the studies.

Several studies have indicated that CD is occurring with increasing prevalence in several geographic regions, particularly in the regions of European origin, indicating that it is generally a lifelong disorder [48]. It usually affects one in a hundred among the general population, being more prevalent in the Middle East and North Africa [13]. The literature demonstrated that the CD prevalence rates were 1% for the United States and Europe and were similar in Argentina and Australia [13]. Prevalence in North Africa has been reported as 0.79% in Libya, 0.6% in Tunisia, and 0.53% in Egypt. A regional study on the Greater Middle East showed a prevalence rate of 0.88% in Iran, while it was 0.6% in Turkey. Studies have shown prevalence in India to be 0.7% [49], whereas in Germany, it was 0.3%, 0.9% in Northern Ireland, 1.2% in Italy, and 2% in Finland [50]. However, in Saudi Arabia, it was between 2.1% and 8.5% [12], and not much statistical data were found on the frequency of CD due to the complexity of diagnosis, not only in adults but also in children.

Many of the studies included in this review concluded that CD occurred more frequently in females and particularly affected children more than adults. Likewise, its adult and childhood occurrence in Sweden was between 1 : 285 and 1: 77 [51], while it was 1 : 230 and 1 : 106 in Italian school-aged kids [52]. Similar trends were also found in non-European peoples such as Australia [53, 54], Argentina [54], Brazil [55], and New Zealand [56]. CD is a female predominant disease having a female to male ratio of 2 : 1 or 3 : 1 [57], which was congruent with the findings of this review. Ciacci et al. [58] established that women were diagnosed with CD at an early age, suffered more symptoms, had lower body mass index according to their age, and had severe anemia. According to Jane Anderson, up to 70% of individuals currently diagnosed with CD are female. This can be explained by two factors. Firstly, more women than men have it, and secondly, women seek medical care more often than males and hence get diagnosed more frequently if they have developed CD.

Despite its increasing prevalence, the diagnosis rate is low. This could be due to poor disease awareness as well as limited diagnostic facilities in these countries. It can sometimes occur as an asymptomatic condition, yet the common gastrointestinal presentations in children include failure to thrive, chronic diarrhea, abdominal distention, and a malabsorptive picture including anorexia, vomiting, and constipation [59]. These symptoms were in line with those evaluated by the majority of the studies where the diagnosis was made only based on the clinical picture and laboratory parameters. Some studies in our review also highlighted the coexistence of CD with other metabolic conditions. Previously published data also supported this comorbidity pattern. From a study, 6% of type 1 diabetes patients while 12% of those with Down syndrome had CD in United States [60]. Along with many other conditions, autoimmune thyroiditis [61] and irritable bowel disease were associated with CD [62].

Most of the studies used anti-tTG titers and EMA for CD diagnosis while one study also showed that the patient underwent endoscopy if any one of the listed tests were positive. Moreover, the anti-tTG was shown to have high specificity and sensitivity in diagnosing CD, specifically in type 1 diabetic children. On a similar note, testing serum levels of anti-tTG was acknowledged as the first choice for CD screening, displaying approximately 98% sensitivity and up to 96% specificity [59]. Improvement of the condition after excluding foods based on gluten products showed improvement in both laboratory indicators and symptoms of the patients. This treatment was also found effective by many other studies [5, 63].

The research review and analysis of this article contributes towards understanding the epidemiology and occurrence of CD in Saudi Arabia as well as other neighboring Middle Eastern and Gulf countries, providing a recent comprehensive overview of the topic. Variation in statistics between the studies can also be attributed to different methodologies and sensitivities of diagnostic tools. There were certain limitations to this review. First, Arabic papers were not included, yet most of the research conducted in Arab countries is published in English anyways. Another important limitation lies in the fact that cross-sectional data cannot be used to infer causality. Another major drawback is that we could not conduct a meta-analysis due to the heterogeneity of data.

## 5. Conclusion

With increasing prevalence, CD is becoming a major public health concern; thus, investigating its epidemiology and clinical features is of great importance. It is now well known that gluten is a precipitating factor, and the research being conducted at present is adding to the understanding of other components of this condition. Awareness of the diversity of presenting symptoms has alerted health professionals to the possible diagnosis of celiac disease. Globally, many patients with celiac disease, including in Saudi Arabia and Gulf countries, remain undiagnosed, which may lead to the development of innovations in screening programs. A growing body of evidence reveals that there is an amplified possibility of celiac disease epidemics soon, especially in the Arab countries that practice gluten-rich dietary patterns. Since many of the cases remain underdiagnosed, the concerned authorities should endeavor to raise the responsiveness of celiac disease. There is a pressing need for research in the future to classify the exact prevalence of the celiac disease.

### 5.1. Implications

Although only 1% of the overall general population has CD, evidence proposed that only around 10% to 15% of this population (children and adults) have been accordingly diagnosed and treated [64]. Hence, early CD diagnosis is crucial, as it might prevent complications. For this to happen, awareness is the key. Emphasis should be on effective communication between the patient and the physician to minimize the disease burden by screening for high-risk individuals. Additionally, periodic follow-up care of such patients is an essential element of effective long-term management of CD. There is a definite need for public health involvement by raising attentiveness towards CD and related dietary habits as well as early screening programs. Future studies in the Arab world should be further aimed at investigating the clinical features of celiac disease and shedding more light on its associated risk factors, preventive measures, early diagnosis, and appropriate treatment modalities. Longitudinal population-based studies are needed in the future to better identify and respond to the burden and risk factors of celiac disease in Arab countries.

## Figures and Tables

**Table 1:** The prevalence of celiac disease among high-risk groups.

S. no.	Authors (year)	Country	Aims	Study design/population	Diagnostic criteria	Results	NOS score
1.	Boudraa et al. (1996) [16]	West Algeria	To assess the prevalence of celiac disease in insulin-dependent diabetes mellitus (IDDM) and explore its presence in their first-degree relatives	Prospective study from 1 January 1993 to 31 December 1994 116 IDDM patients 381 first-degree relatives of IDDM patients	Serological markers, IgA and IgG antigliadin antibodies (AGA), and IgA antiendomysium antibodies (EMA) Jejunal biopsy of symptomatic patients	Prevalence of CD in IDDM patients was 16% to 20% (since not all patients with positive serological markers experienced jejunal biopsy, the prevalence can be considerably higher up to 20%) In 1^st^-degree relatives, 6.8% positive for one serological marker, while 3.4% had villous atrophy.	6
2.	Al Attas (2002) [17]	Eastern Saudi Arabia	To estimate CD prevalence in clinically suspicious celiac disease patients and in patients with disorders considered to have an association with CD, such as autoimmune diseases	Hospital-based study Group 1 =145 patients (clinically suspected) Group 2 = 80 with autoimmune diseases Group 3 = 20 patients with IBD Group 4 = 100 heathy blood donors	IgA-EMA and intestinal biopsy of confirmed cases	*Group* 1 = *EMA*‐*positive* 7.6%, *biopsy* *confirmed* = 4% *group* 2 = *EMA*‐*positive* 2.5% (all have autoimmune thyroid disease (AITD)), and *groups* 3 and 4 = *no* *positive* *EMA*.	7
3.	Ashabani et al. (2003) [18]	Libya	To investigate the CD-related marker occurrence in Libyan children patients with DM	Cohort study conducted on 234 Libyan children with DM (age range 2 to 25 years) and 50 healthy children	IgA and IgG, AGA, anti-tTG, anticalreticulin antibodies, and EMA	50 (21.3%) positive for IgA and/or IgG-AGA, tTG, and anticalreticulin antibodies 19 of these were EMA positive 24 had biopsy-proven CD including EMA-negative patient with IgA deficiency Overall, CD prevalence found 10.3%	6
4.	Al-Ashwal et al. (2003) [19]	Saudi Arabia	To examine celiac disease prevalence in young Saudi patients suffering from type I diabetes mellitus at “King Faisal Specialist Hospital and Research Centre, Riyadh”	Hospital-based research; 123 type 1 diabetic patients	Serum gliadin immunoglobulin (Ig) A and reticulin IgA antibody	Serology positive 10 (8.1%) 6 had jejunal biopsy and showed villus atrophy; thus, prevalence was 4.9%, based on biopsy results and antibodies.	7
5.	Nowier et al. (2009) [20]	Egypt	Celiac disease prevalence among Egyptians with type 1 diabetes and the association with autoimmune thyroid disease	Case-control study design where case and control groups were compared 73 type 1 DM patients	Enzyme-linked immunosorbent assay (ELISA) antibodies to tTG	Prevalence of CD among type 1 DM patients was 5.48% positive anti-tTG antibodies Anti-tTG antibody testing was negative for patients with autoimmune thyroid disease.	8
6.	Al-Hussaini et al. (2012) [21]	Middle East	To identify the epidemiology of celiac diseases among type 1 diabetes in Middle Eastern children	Cross-sectional study; 106 children with type 1 diabetes	IgA anti-tTG and EMA	19 (18%) children showed positive results of anti-tTG and/or EMA 12 (11.3%) children were found to be CD positive by biopsy.	6
7.	Saadah et al. (2012) [22]	Saudi Arabia	CD prevalence in adolescent and children patients with type 1 DM	Retrospective hospital record-based study 430 diabetic children	Anti-tTG antibodies	91 (21.2%) positive for anti-tTG antibody 48 (11.2%) patients' biopsy confirmed CD (42 asymptomatic).	3
8.	Al-Sinani et al. (2013) [23]	Oman	Celiac disease prevalence in Omani children (type 1 diabetics)	A prospective cross-sectional study 103 children with type 1 diabetes	Anti-tTG IgA, EMA IgA, and total IgA Endoscopy and biopsy	17% (*N* = 14) positive anti-tTG 5.5% (*n* = 5) biopsy proven CD. Among these all 5 were also positive for EMA.	5
9.	Farahid et al. (2014) [11]	Jordan	To estimate celiac disease prevalence in AIH patients in Jordan and to determine patients at higher disease risk	Cross-sectional record-based review; 914 AIH patients (108 males and 806 females) aged 20–82 years	EMA IgA and IgG Duodenal biopsy	117 (12.8%) seropositive for CD. 39 (44.8%) out of 87 biopsy proven CD prevalence among patients with AIH was estimated to be 5.7% in comparison to seroprevalence of 12.8% Higher association was found between CD and *age* > 40 years, vitamin B12 deficiency, anemia, and other autoimmune diseases for example, Addison disease, diabetes mellitus, and vitiligo.	6
10.	Al-Hakami (2016) [24]	Saudi Arabia	To determine the seroprevalence of coexisting autoantibodies among patients with type 1 diabetes and to look for possible association with glycemic control, diabetes duration, and diagnosis at Aseer Central Hospital, Abha	Cross-sectional study 202 T1DM patients were included in this study	Anti-tTG, EMA	21 (10.4%) positive for both anti-tTG and EMA No significant association between the age at T1DM glycemic control, duration, and diagnosis and the autoantibody presence was observed.	5
11.	Al-Ajlan (2016) [25]	Saudi Arabia	To examine the implications and prevalence of celiac disease among Saudi adults and comparing it with diagnosed with irritable bowel syndrome at Al-Iman General Hospital and Prince Salman Hospital, Riyadh	Prospective case-control study Subjects aged 20-60 980 adult patients Among them, 482 subjects were controls and 498 with IBS	Anti-tTG and EMA and biopsy	1.9% CD in control group 9.6% in IBS group 55 out of 980 patients were found to be positive for celiac disease.	8
12.	Al-Hakami (2016) [26]	Saudi Arabia	To estimate the prevalence of CD in high-risk groups in Aseer (southwest region) and to determine its associations	Laboratory records (retrospective case-finding) 315 patients	Anti-tTG and EMA and biopsy	58 (18.4%) got a positive test for at least one antibody marker 17.5% positive for anti-tTG 15.6% positive for EMA 22 out of 40 biopsies were confirmed for CD Type 1 DM was the most common clinical illness related to these markers with the percentage 47% However, gastrointestinal presentations were observed to be only 11.5%.	4
13.	Mansour and Najeeb (2011) [27]	Iraq	To evaluate silent CD frequency in Iraqi patients' sample with type 1 diabetes mellitus	Prospective cross-sectional from November 2008 to December 2009; 62 patients with type 1 diabetes mellitus from age 8 to 42	IgA, anti-tTG-IgA, anti-tTG-IgG, EMA-IgG, and duodenal biopsy	11.2% in Iraqi patients with type 1 DM. 43.55% had Marsh 0 16.1% had Marsh I 0% had Marsh II 3.2% had Marsh IIIA 4.83% Marsh IIIB 3.2% Marsh IIIC For diagnostic purposes, EMA and tTG tests were found to be useful.	6
14.	Fraser et al. (2003) [28]	Oman	To study the association between occult celiac disease and iron deficiency anemia in Omani adults in Sultan Qaboos University Hospital, Muscat	Hospital-based study 51 patients	IgA, anti-tTG-IgA, anti-tTG-IgG, EMA-IgG, and duodenal biopsy	Mean Hb 9 with confirmed low ferritin. 2 patients positive IgA-tTG and IgA EMA and IgG tTG One patient biopsy done and showed villous atrophy. Prevalence considered being approximately 1 : 30 in iron deficiency patients and 1 in 200-300 affected in the general population.	4
15.	Oujamaa et al. (2019) [29]	Morocco	To examine the prevalence of specific autoantibodies to CD in adult and pediatric population with type 1 diabetes	Multicenter, cross-sectional study Study population consists of 276 adults and pediatric diabetic patients	Anti-tTG-IgA, anti-tTG-IgG, EMA, HLA-DQ2/DQ8 typing, and duodenal biopsy	Seroprevalence of CD in T1D patients was 9.1% (CI = 95%) 2 cases had biopsy-proven CD.	5
16	Alyafei et al. (2018) [30]	Qatar	To determine the prevalence of autoantibodies in diabetic patients in Qatar	Retrospective cross-sectional study, 490 pediatric patients aged 0.5-16 years	Anti-tTG IgA and anti-tTG IgG Biopsy	In 365 T1DM, 18 (5%) patients have positive anti-tTG IgA and 16 (4.3%) anti-tTG IgG antibodies. In 46 T2DM, anti-tTG IgA antibodies were found in 4 patients (8.7%), whereas no anti-tTG IgG antibodies detected in any patient. Mucosal biopsy proved celiac disease in 9 out of 12 patients (75%) with positive ATT IgA and IgG antibodies.	4
17	Odeh et al. (2019) [31]	Jordan	To determine the prevalence of biopsy-proven CD among T1DM pediatric patients	Mixed prospective and retrospective study 538 children with T1DM Data collected from 2012 to 2017	IgA-tTG and IgG-tTG antibodies Duodenal biopsy	Prevalence of serology positive CD was 16.6% while biopsy-proven CD was 9.1%.	5
18	AlRuwaily et al. (2017) [32]	Saudi Arabia	To determine the prevalence of CD in Down syndrome Saudi patients	Retrospective study, files of 91 pediatric patients for serological markers and biopsy results	Antigliadin antibody (AGA) IgA and IgG, EMA, IgA-tTG, and IgG-tTG antibodies	(i) AGA-IgA found in 32.14% (ii) AGA IgG in 52.38% (iii) EMA tested positive in 14.28% and negative in 69.04% (iv) Anti-tTG IgA was high in 15.5% (v) Serum IgA normal level found in 43% patients while low in 1.2%. Biopsy-confirmed cases of CD was 10.7%.	5
19	Alghamdi et al. (2018) [33]	Saudi Arabia	To determine the prevalence of CD in T1DM patient living in Al-Baha region, Saudi Arabia	Retrospective record-based study 268 T1DM patients of age 2-23 years	IgA-tTG and IgG-tTG antibodies	Prevalence of serology positive cases of CD was 7.1%..	3
20	Alshareef et al. (2016) [34]	Saudi Arabia	To determine the prevalence of CD in T1DM patient of Saudi Arabia	Cross-sectional study 218 T1DM patients with *age* ≥ 12 *years*	Anti-tTG antibodies and duodenal biopsy	Raised anti-TTG levels found in 7.3% patients. Duodenal biopsies were done in 12 patients which showed (i) total villous atrophy 3.7% (ii) subtotal villous atrophy 0.8% Chronic duodenitis 0.8%	4
21	Al-Agha et al. (2015) [35]	Saudi Arabia	To investigate the coexistence of autoimmune diseases in T1DM patients	Cross-sectional study 228 patients with age 1-18 years	Anti-tTG antibodies and jejunal biopsy	Celiac disease was found in 19.7%. CD was also significantly associated with a high level of HbA1C level (OR = 1.016; 95% CI: 0.884-1.166).	5
22	Abdulrazzaq et al. (2018) [36]	UAE	To investigate the presence of autoimmune diseases in Emirati children with Down's syndrome	Cross-sectional study conducted on 92 Down's syndrome patients	Anti-tTG antibodies	Prevalence of CD in study population was 1.1%.	3

Abbreviation: IgA-tTG: antitissue transglutaminase IgA; IgG-tTG: antitissue transglutaminase IgG; EMA: antiendomysium antibodies; AGA: antigliadin antibodies; NOS: Newcastle-Ottawa Scale.

**Table 2:** The prevalence of celiac disease among general population.

S. no.	Authors (year)	Country	Aims	Study design/population	Diagnostic criteria	Results	NOS score
1.	Bdioui et al. (2006) [8]	Tunisia	To determine CD prevalence among Tunisian healthy blood donors	Prospective study; total 1418, 1090 men and 328 women	IgA-EMA, anti-tTG, and biopsy	Prevalence of CD was about 1/700 among blood donors 3 positives for IgA EMA, where 2 were positive for anti-tTG and also showed villous atrophy	5
2.	Hariz et al. (2007) [37]	Tunisia	To determine CD prevalence among Tunisian children and to describe the clinical profile of the screened patients	Mass screening study; 6286 children	IgA-tTG, IgA-AE, and biopsy	139 positives for IgA-tTG 40 positives for IgA-AE 28 had positive for both (IgA-tTG, IgA-AE); biopsy-proven CD found in 26 participants 79 had positive test for only IgA-tTG; among them, biopsy was normal Estimated prevalence in school children 1/157.	5
3.	Khayyat (2012) [38]	Western region of Saudi Arabia	Gluten sensitivity prevalence in healthy Saudi adults at “King Faisal Specialist Hospital & Research Centre in Jeddah, Saudi Arabia”	Prospective pilot research for Saudi attendees (in blood donation center); 204 individuals (122 males and 82 females)	Anti-tTG IgA and IgA level	3 (1.5%) people tested positive for IgA TTG showing normal IgA level.	4
4.	Aljebreen et al. (2013) [39]	Saudi Arabia	To recognize the seroprevalence of CD among healthy adolescents in Saudi Arabia	Quantitative research by randomly selecting 10th- to 12th-grade students from 3 distinct Saudi regions, including Al-Qaseem, Madinah, and Aseer 1167 students	EMA and IGA by indirect immunofluorescence	2.2% (26 students) showed a positive anti-EMA test The prevalence was highest in the Al-Qaseem region (3.2%) However, the lowest prevalence was found in Madinah (1.8%).	6
5.	Al-Hussaini et al. (2017) [14]	Saudi Arabia	To determine celiac disease (CD) prevalence and illustrate the iceberg of celiac disease among Saudi pediatric population in Riyadh	Prospective cross-sectional study 7930 students	Anti-tTG IgA and EMA-IgA and biopsy	221 (2.8%) students with positive TTG-IgA, CD diagnosed in 119 cases. High CD prevalence among Saudi children was estimated to be 1.5%.	5
6.	Al Hatlani (2015) [40]	Saudi Arabia	To determine the prevalence of CD among symptom-free children from the military campus (public school) of National Guard in the Eastern Province, Saudi Arabia	Cross-sectional study 1141 students	Anti-tTG-IgA and IgG antibodies and intestinal biopsy	32 (3%) IgA-tTG positive An intestinal biopsy was also undertaken in 10 of them 1% biopsy-confirmed prevalence.	5
7.	Abu-Zekry et al. (2008) [41]	Egypt	To examine celiac disease frequency in Egyptian children	Prospective cross-sectional study Group A: 1500 general pediatric population Group B: 150 admitted patient with diarrhea and failure to thrive Group C: 250with T1DM	Anti-tTG, IgA EMA, total IgA, IgG anti-tTG Small bowel biopsy	CD diagnosis was made in 2 groups of patients: A and B Group A: 8 children diagnosed with CD (1 in 187 individuals (0.53%; 95% CI 0.17%–0.89%)). Group B: 7 had CD (4.7%, 95% CI 1.4–7.9) Group C: 16 serology-positive CD (6.4%; 95% CI 3.4–9.4).	7
8.	Mankai et al. (2006) [42]	Tunisia	To screen CD in healthy blood donors in Tunisia	Retrospective cross-sectional study, serological screening of 2500 healthy blood donors	IgG-AGA, IgA-AGA, and EMA	418 samples were positive for AGA, 7 of them tested positive for AEA (which had amplified IgA and/or IgG AGA levels) The prevalence of EMA was 1 : 355.	5
9.	Abu-Zeid et al. (2014) [43]	United Arab Emirates	Celiac disease prevalence in healthy UAE national adolescents	Quantitative research Cross-sectional prospective research 1197 healthy Emiratis	Anti-tTG IgA antibodies and EMA IgA antibodies	1.17% seropositive for anti-tTG IgA and EMA IgA antibodies. The seroprevalence of CD was found to be 1 : 86 among adult UAE nationals (1 : 624 for men) and (1 : 44 for women). A higher frequency of CD among women as compared to men.	7

Abbreviation: IgA-tTG: antitissue transglutaminase IgA; IgG-tTG: antitissue transglutaminase IgG; EMA: antiendomysium antibodies; AGA: antigliadin antibodies; NOS: Newcastle-Ottawa Scale.

**Table 3:** Clinical characteristics in clinically and laboratory CD-confirmed population.

S. no.	Authors (year)	Country	Aims	Study design/population	Diagnostic criteria	Case definition	NOS score
1	Wafa'a Al-Qabandi et al. (2015) [44]	Kuwait	To share the experience of dealing with Kuwaiti children suffering from celiac disease	Retrospective research 47 patients of CD serology and biopsy proven (symptomatic: 25, screened: 22) Age range from 7 to 189 months	EMA, AGA-IgA, AGA-IgG, and anti-tTG	66% females, 34% males, 85% EMA positive, 79% AGA-IgA positive, and 77% AGA-IgG positive. 19 have T1D, 2 have Down's syndrome, 1 has both T1D and Down's syndrome, 3 have hypothyroidism, and 1 juvenile has idiopathic arthritis 9% had celiac disease family history.	6
2	Saadah (2011) [45]	Saudi Arabia	To identify the clinical pattern of celiac disease prevalence	Retrospective, hospital-based research	Anti-tTG, IgA, IgG antibodies and biopsy proven	80 children were diagnosed with celiac disease (age range of 0.5–18 years) 39 (49%) individuals showed conventional symptoms of malabsorption, while 41 (51%) were found to be at high risk of developing CD. 73 (91%) = positive anti‐tTG antibodies 18 (23%) = positive IgG antibodies 46 (58%) = positive IgA antibodies 11 out of 65 individuals showed disturbed liver function tests.	5
3	Sarkhy et al. (2015) [46]	Saudi Arabia	To address clinical characteristics of celiac disease among Saudi children as well as to examine the adherence rate to gluten-free diet along with its determinant factors	Cross-sectional study 113 children; median age 9.9 years	Biopsy-confirmed cases	92% of the patients were symptomatic while 8% were asymptomatic. Out of total, 62 of the children were females. The most commonly presenting symptoms include poor weight gain (54%), chronic abdominal pain (59.3%), abdominal distention, gases, bloating (46.1%), and chronic diarrhea (41.6%). Shorter duration since the diagnosis and younger age at diagnosis were interrelated with an improved adherence rate.	7
4	Saeed et al. (2017) [47]	Saudi Arabia	To characterize the clinical presentations and diagnosis in children under the age of 18 with celiac disease at a private tertiary care health care center in Riyadh	Retrospective study 59 children	IgA-tTG and IgG-tTG antibodies and biopsy	50.8% males Median age 8 years Mean duration of symptoms before diagnosis 2.3 years (±1.5). Classical disease was merely observed in 30.5%, while 69.5% had either nonclassical presentations or belonged to high-risk groups for celiac disease 91.5% positive for IgA-tTG antibodies 81.3% positive for IgG-tTG 52 had Marsh grade III lesion.	5

Abbreviation: IgA-tTG: antitissue transglutaminase IgA; IgG-tTG: antitissue transglutaminase IgG; EMA: antiendomysium antibodies; AGA: antigliadin antibodies; NOS: Newcastle-Ottawa Scale.

## References

[1] Cohen I. S., Day A. S., Shaoul R. (2019). Gluten in celiac disease–more or less?. *Rambam Maimonides Medical Journal*.

[2] Assa A., Frenkel-Nir Y. (2017). Anthropometric measures and prevalence trends in adolescents with coeliac disease: a population based study. *Archives of Disease in Childhood*.

[3] Ludvigsson J. F., Leffler D. A., Bai J. C. (2013). The Oslo definitions for coeliac disease and related terms. *Gut*.

[4] Husby S., Koletzko S., Korponay-Szabo I. (2012). European Society for Pediatric Gastroenterology, Hepatology, and Nutrition guidelines for the diagnosis of coeliac disease. *Journal of Pediatric Gastroenterology and Nutrition*.

[5] Barada K., Bitar A., Mokadem M. A.-R., Hashash J. G., Green P. (2010). Celiac disease in Middle Eastern and North African countries: a new burden?. *World Journal of Gastroenterology*.

[6] Kang J., Kang A., Green A., Gwee K., Ho K. (2013). Systematic review: worldwide variation in the frequency of coeliac disease and changes over time. *Alimentary Pharmacology & Therapeutics*.

[7] Fasano A., Berti I., Gerarduzzi T. (2003). Prevalence of celiac disease in at-risk and not-at-risk groups in the United States: a large multicenter study. *Archives of Internal Medicine*.

[8] Bdioui F., Sakly N., Hassine M., Saffar H. (2006). Prevalence of celiac disease in Tunisian blood donors. *Gastroentérologie clinique et biologique*.

[9] Irvine A. J., Chey W. D., Ford A. C. (2017). Screening for celiac disease in irritable bowel syndrome: an updated systematic review and meta-analysis. *The American Journal Of Gastroenterology*.

[10] Hamzeh A., Nair P., Al-Khaja N., Al A. M. (2015). Association of HLA-DQA1 and -DQB1 alleles with type I diabetes in Arabs: a meta-analyses. *Tissue Antigens*.

[11] Farahid O., Khawaja N., Shennak M., Batieha A., El Khateeb M., Ajlouni K. (2014). Prevalence of coeliac disease among adult patients with autoimmune hypothyroidism in Jordan. *Eastern Mediterranean Health Journal*.

[12] Singh P., Arora S., Singh A., Strand T. A., Makharia G. K. (2016). Prevalence of celiac disease in Asia: a systematic review and meta-analysis. *Journal of Gastroenterology and Hepatology*.

[13] Catassi C. (2017). New celiac icebergs are spotted, other are slowly emerging. *Journal of Pediatric Gastroenterology and Nutrition*.

[14] Al-Hussaini A., Troncone R., Khormi M. (2017). Mass screening for celiac disease among school-aged children: toward exploring celiac iceberg in Saudi Arabia. *Journal of Pediatric Gastroenterology and Nutrition*.

[15] Peterson J., Welch V., Losos M., Tugwell P. J. (2011). *The Newcastle-Ottawa Scale (NOS) for Assessing the Quality of Nonrandomised Studies in Meta-Analyses*.

[16] Boudraa G., Hachelaf W., Benbouabdellah M., Belkadi M., Benmansour F., Touhami M. (1996). Prevalence of coeliac disease in diabetic children and their first-degree relatives in West Algeria: screening with serological markers. *Acta Paediatrica*.

[17] Al Attas R. A. (2002). How common is celiac disease in Eastern Saudi Arabia. *Annals of Saudi Medicine*.

[18] Ashabani A., Abushofa U., Abusrewill S., Abdelazez M., Tučková L., Tlaskalová-Hogenová H. (2003). The prevalence of coeliac disease in Libyan children with type 1 diabetes mellitus. *Diabetes/Metabolism Research and Reviews*.

[19] Al-Ashwal A. A., Shabib S. M., Sakati N. A., Attia N. A. (2003). Prevalence and characteristics of celiac disease in type I diabetes mellitus in Saudi Arabia. *Saudi Medical Journal*.

[20] Nowier S. R., Eldeen N. S., Farid M. M., Rasol H., Mekhemer S. M. (2009). Prevalence of celiac disease among type 1 diabetic Egyptian patients and the association with autoimmune thyroid disease. *Bratislavské Lekárske Listy*.

[21] Al-Hussaini A., Sulaiman N., Al-Zahrani M., Alenizi A., El Haj I. (2012). High prevalence of celiac disease among Saudi children with type 1 diabetes: a prospective cross-sectional study. *BMC Gastroenterology*.

[22] Saadah O. I., Al-Agha A. E., Al Nahdi H. M. (2012). Prevalence of celiac disease in children with type 1 diabetes mellitus screened by anti-tissue transglutaminase antibody from Western Saudi Arabia. *Saudi Medical Journal*.

[23] Al-Sinani S., Sharef S. W., Al-Yaarubi S. (2013). Prevalence of celiac disease in Omani children with type 1 diabetes mellitus: a cross sectional study. *Oman Medical Journal*.

[24] Al-Hakami A. M. (2016). Pattern of thyroid, celiac, and anti-cyclic citrullinated peptide autoantibodies coexistence with type 1 diabetes mellitus in patients from southwestern Saudi Arabia. *Saudi Medical Journal*.

[25] Al-Ajlan A. S. (2016). Screening of coeliac disease in undetected adults and patients diagnosed with irritable bowel syndrome in Riyadh, Saudi Arabia. *Saudi Journal Of Biological Sciences*.

[26] Al-Hakami A. M. (2016). Seroprevalence of coeliac disease in at-risk subjects at the main tertiary hospital, southwest of Saudi Arabia. *Arab Journal of Gastroenterology*.

[27] Mansour A. A., Najeeb A. A. (2011). Coeliac disease in Iraqi type 1 diabetic patients. *Arab Journal of Gastroenterology*.

[28] Fraser J., Woodhouse N. J., El-Shafie O., Al-Kindy S., Ciclitira P. (2003). Occult celiac disease in adult Omanis with unexplained iron deficiency anemia. *Saudi Medical Journal*.

[29] Oujamaa I., Sebbani M., Elmoumou L. (2019). The prevalence of celiac disease-specific auto-antibodies in type 1 diabetes in a Moroccan population. *International Journal of Endocrinology*.

[30] Alyafei F., Soliman A., Alkhalaf F. (2018). Prevalence of *β*-cell antibodies and associated autoimmune diseases in children and adolescents with type 1 diabetes (T1DM) versus type 2 diabetes (T2DM) in Qatar. *Acta bio-medica: Atenei Parmensis*.

[31] Odeh R., Alassaf A., Gharaibeh L., Ibrahim S., Khdair F., Ajlouni K. (2019). Prevalence of celiac disease and celiac-related antibody status in pediatric patients with type 1 diabetes in Jordan. *Endocrine Connections*.

[32] AlRuwaily F., Kattan H. A., AlMehaidib A. M., AlDekhail W. (2017). Prevalence of celiac disease in Saudi children with Down syndrome: a retrospective study. *International Journal of Pediatrics and Adolescent Medicine*.

[33] Alghamdi R. A., Alghamdi A. H., Fureeh A. A. (2018). Sero-prevalence of celiac disease among symptom-free type 1 diabetes mellitus in Al-Baha region, Saudi Arabia. *Journal of Pharmacy and Biological Sciences*.

[34] Alshareef M., Aljabri K., Bokhari S., Al Jiffri A., Abu Elsaoud H., Akl A. (2016). The prevalence of celiac disease in Saudi patients with type 1 diabetes mellitus: cross sectional study. *International Journal of Diabetes Metabolic Disorders*.

[35] Al-Agha A., Alafif M., Abd-Elhameed I. (2015). Glycemic control, complications, and associated autoimmune diseases in children and adolescents with type 1 diabetes in Jeddah, Saudi Arabia. *Saudi Medical Journal*.

[36] Abdulrazzaq Y., El-Azzabi T. I., Al Hamad S. M., Attia S., Deeb A., Aburawi E. H. (2018). Occurrence of hypothyroidism, diabetes mellitus, and celiac disease in Emirati children with Down’s syndrome. *Oman Medical Journal*.

[37] Hariz M. B., Kallel-Sellami M., Kallel L. (2007). Prevalence of celiac disease in Tunisia: mass-screening study in schoolchildren. *European Journal Of Gastroenterology & Hepatology*.

[38] Khayyat Y. M. (2012). Serologic markers of gluten sensitivity in a healthy population from the western region of Saudi Arabia. *Saudi Journal of Gastroenterology*.

[39] Aljebreen A. M., Almadi M. A., Alhammad A., Al Faleh F. Z. (2013). Seroprevalence of celiac disease among healthy adolescents in Saudi Arabia. *World Journal of Gastroenterology*.

[40] Al Hatlani M. M. (2015). Prevalence of celiac disease among symptom-free children from the Eastern Province of Saudi Arabia. *Saudi Journal of Gastroenterology*.

[41] Abu-Zekry M., Kryszak D., Diab M., Catassi C., Fasano A. (2008). Prevalence of celiac disease in Egyptian children disputes the east–west agriculture-dependent spread of the disease. *Journal of Pediatric Gastroenterology and Nutrition*.

[42] Mankai A., Landolsi H., Chahed A. (2006). Celiac disease in Tunisia: serological screening in healthy blood donors. *Pathologie Biologie*.

[43] Abu-Zeid Y. A., Jasem W. S., Lebwohl B., Green P. H., ElGhazali G. (2014). Seroprevalence of celiac disease among United Arab Emirates healthy adult nationals: a gender disparity. *World Journal of Gastroenterology*.

[44] Wafa’a Al-Qabandi E. B., Al-Abdulrazzaq D., Hamadi K., Al R. F. (2015). Celiac disease in children: is it a problem in Kuwait?. *Clinical and Experimental Gastroenterology*.

[45] Saadah O. I. (2011). Celiac disease in children and adolescents at a singe center in Saudi Arabia. *Annals of Saudi Medicine*.

[46] Al Sarkhy A., El Mouzan M. I., Saeed E. (2015). Clinical characteristics of celiac disease and dietary adherence to gluten-free diet among Saudi children. *Pediatric Gastroenterology, Hepatology & Nutrition*.

[47] Saeed A., Assiri A., Assiri H., Ullah A., Rashid M. (2017). Celiac disease in Saudi children: evaluation of clinical features and diagnosis. *Saudi Medical Journal*.

[48] Lionetti E., Gatti S., Pulvirenti A., Catassi C. (2015). Celiac disease from a global perspective. *Best Practice & Research Clinical Gastroenterology*.

[49] Lionetti E., Catassi C. (2011). New clues in celiac disease epidemiology, pathogenesis, clinical manifestations, and treatment. *International Reviews of Immunology*.

[50] Mustalahti K., Catassi C., Reunanen A. (2010). The prevalence of celiac disease in Europe: results of a centralized, international mass screening project. *Annals of Medicine*.

[51] Carlsson A. K., Axelsson I. E., Borulf S. K., Bredberg A. C., Ivarsson S.-A. (2001). Serological screening for celiac disease in healthy 2.5-year-old children in Sweden. *Pediatrics*.

[52] Tommasini A., Not T., Kiren V. (2004). Mass screening for coeliac disease using antihuman transglutaminase antibody assay. *Archives of Disease in Childhood*.

[53] Hovell C. J., Collett J. A., Vautier G. (2001). High prevalence of coeliac disease in a population-based study from Western Australia: a case for screening?. *Medical Journal of Australia*.

[54] Hovell C. J., Collett J. A., Vautier G. (2001). Prevalence of celiac disease in Argentina: screening of an adult population in the La Plata area. *The American Journal of Gastroenterology*.

[55] Oliveira R. P., Sdepanian V. L., Barreto J. A. (2007). High prevalence of celiac disease in Brazilian blood donor volunteers based on screening by IgA antitissue transglutaminase antibody. *European Journal of Gastroenterology & Hepatology*.

[56] Cook H. B., Burt M. J., Collett J. A., Whitehead M. R., Frampton C. M., Chapman B. A. (2001). Adult coeliac disease: prevalence and clinical significance. *Journal of Gastroenterology and Hepatology*.

[57] Bai D., Brar P., Holleran S., Ramakrishnan R., Green P. H. (2009). Effect of gender on the manifestations of celiac disease: evidence for greater malabsorption in men. *Scandinavian Journal of Gastroenterology*.

[58] Ciacci C., Cirillo M., Giorgetti G. (1999). Low plasma cholesterol: a correlate of nondiagnosed celiac disease in adults with hypochromic anemia. *The American Journal of Gastroenterology*.

[59] Guandalini S., Assiri A. (2014). Celiac disease: a review. *JAMA Pediatrics*.

[60] Rampertab S. D., Pooran N., Brar P., Singh P., Green P. H. (2006). Trends in the presentation of celiac disease. *The American Journal of Medicine*.

[61] Lerner A., Jeremias P., Matthias T. (2017). Gut-thyroid axis and celiac disease. *Endocrine Connections*.

[62] El-Salhy M., Hatlebakk J. G., Gilja O. H., Hausken T. (2015). The relation between celiac disease, nonceliac gluten sensitivity and irritable bowel syndrome. *Nutrition Journal*.

[63] Amiriani T., Besharat S., Roshandel G., Shalizar A. (2011). Should we look for celiac disease in irritable bowel syndrome?. *Oman Medical Journal*.

[64] Rubio-Tapia A., Ludvigsson J. F., Brantner T. L., Murray J. A., Everhart J. E. (2012). The prevalence of celiac disease in the United States. *The American Journal Of Gastroenterology*.

